# Persistent Left Superior Vena Cava Draining into the Coronary Sinus: A Case Report

**DOI:** 10.4021/cr85w

**Published:** 2011-09-20

**Authors:** Ertugrul Kurtoglu, Ozlem Cakin, Selahaddin Akcay, Erdal Akturk, Hasan Korkmaz

**Affiliations:** a Elazig Training and Research Hospital, Clinic of Cardiology, Elazig, Turkey; b Harput State Hospital, Clinic of Internal Medicine, Elazig, Turkey; c Harput State Hospital, Clinic of Cardiology, Elazig, Turkey; d Adiyaman University, Department of Cardiology, Adiyaman, Turkey

**Keywords:** Vena cava superior, Echocardiography, Coronary sinus

## Abstract

Persistent left superior vena cava (PLSVC) is a congenital anomaly of the thoracic venous system resulting from the abnormal persistence of an embryological vessel that normally regresses during early fetal life. This anomaly is often discovered incidentally during surgery, cardiovascular imaging or invasive cardiovascular procedures. In most cases, a PLSVC drains into the right atrium through the coronary sinus. In the remainder of cases, it enters directly or through the pulmonary veins into the left atrium. A dilated coronary sinus on echocardiography should always raise the suspicion of a PLSVC as it has important clinical implications. The diagnosis should be confirmed by saline contrast echocardiography. We report a patient with persistent left superior vena cava with an enlarged coronary sinus and normal right superior vena cava.

## Introduction

Persistent left superior vena cava (PLSVC) is a rare venous abnormality. It is, however, the most common congenital anomaly of thoracic venous system with a frequency of less than 0.5% of the general population and up to 10% of patients with congenital heart disease [1-3]. It is usually asymptomatic and an incidental finding often detected during cardiovascular imaging [4-6], surgery [7], left subclavian vein cannulation [8-10] or device implantation [11, 12]. PLSVC is the result of persistent patency of left cardinal vein that is present in the early stages of fetal life. In the most of cases, it drains into right atrium through the coronary sinus in the absence of congenital heart disease [13]. In the remainder of cases, drainage to the left atrium occurs because of failure to form the coronary sinus and it is associated with many types of congenital heart disease [14, 15].

## Case Report

**Figure 1:**
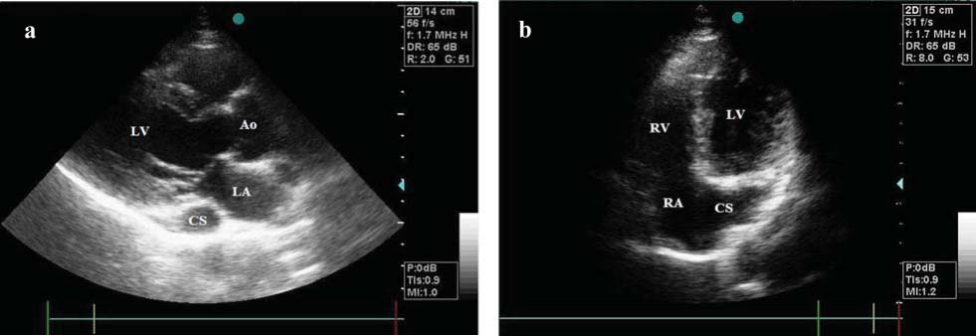
(a)Transthoracic echocardiogram in parasternal long-axis view illustrating a dilated coronary sinus; (b)Transthoracic echocardiogram in apical right ventricular inflow view illustrating a dilated coronary sinus. (Ao: aorta; CS: coronary sinus; LA: left atrium; LV: left ventricle; RA: right atrium; RV: right ventricle)

**Figure 2:**
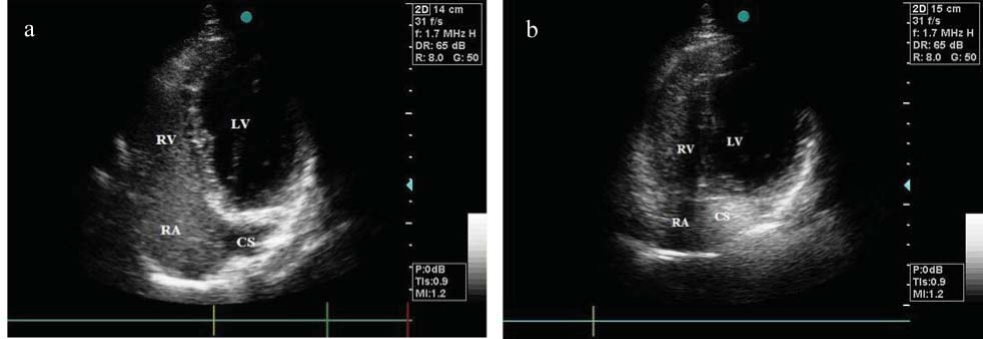
(a)Transthoracic echocardiogram in apical right ventricular inflow view illustrating early opacification of right chambers following agitated saline injection from right antebrachial vein; (b)Transthoracic echocardiogram in apical right ventricular inflow view illustrating early opacification of the abnor­mally large coronary sinus following agitated saline injection from left antebrachial vein. (CS: coronary sinus; LV: left ventricle; RA: right atrium; RV: right ventricle)

**Figure 3:**
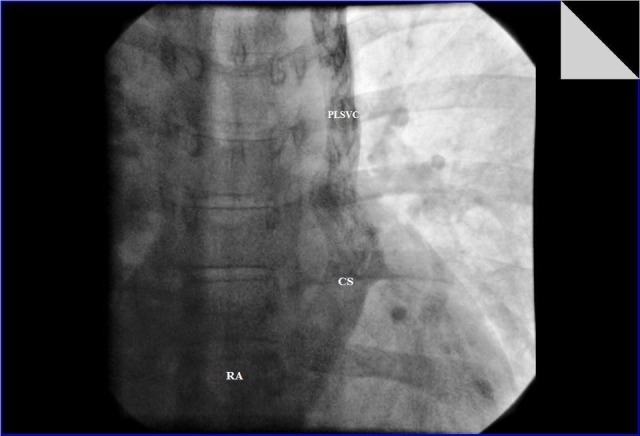
Fluoroscopic image of agitated saline injection showing persistent left superior vena cava draining into right atrium through the coronary sinus. (PLSVC: persistent left superior vena cava; CS: coronary sinus; RA: right atrium)

A 34-year-old female was referred by her primary physican for echocardiographic evaluation because of diastolic murmur. The patient did not have any symptom or a history of cardiovascular disease. Physical examination was unremarkable except a mild diastolic murmur at the lower left sternal border. A transthoracic echocardiography revealed a dilated coronary sinus (CS) with normal left and right cavities (Fig. 1a, b). Injection of agitated saline from patient’s left antecubital vein resulted in early opacification of the coronary sinus before other right-side chambers, thus suggesting the presence of a PLSVC draining to the coronary sinus. When agitated saline was injected into patient’s right antecubital vein, bubble contrast appeared first in the right atrium, which was considered evidence of a normal right superior vena cava (Fig. 2a, b). For further confirmation of PLSVC, a left brachial venogram was performed using 20 cc of nonionic contrast injected into the left antecubital vein. The cineangiogram demonstrated a PLSVC that drained vertically downward into a dilated coronary sinus (Fig. 3). As the diagnosis was well-established by fluoroscopy, further imaging modalities were deemed unnecassary.

## Discussion

PLSVC is an uncommon and yet the most commonly reported thoracic venous abnormality. The frequency of a PLSVC is 0.3-0.5% among healthy individuals and as many as 10% of patients with congenital heart diseases [1-3].

The thoracic embryonic venous system is composed of anterior cardinal veins which drain the cephalic part of the embryo and posterior cardinal veins which drain the caudal part of embryo. Anterior and posterior veins join to form the short common cardinal veins before entering the embryological heart. During the eighth week of fetal life, the anterior cardinal veins become connected by an anastomosis. This anastomosis shunts blood from the left to the right anterior cardinal vein and ultimately becomes innominate (or brachiocephalic) vein when the caudal part of the left anterior cardinal vein regresses to become “ligament of Marshall”. If this regression does not occur, a left-sided vascular structure which drains to the right atrium through the coronary sinus will persist. The cephalic parts of anterior cardinal veins form the internal jugular veins and the caudal part of the right anterior cardinal vein develop into the normal right superior vena cava (RSVC) [16].

There are two types of PLSVC described in the literature. In 92% of cases, PLSVC connects to the right atrium via coronary sinus with no hemodinamically significant consequence and in 8% of cases, PLSVC connects directly or through the pulmonary veins to the left atrium causing a right to left shunt [17-20]. Almost 40% of patients, PLSVC is accompanied by a variety of cardiac anomalies such as atrial septal defect, bicuspid aortic valve, cor triatrium and coarctation of aorta [21]. In 80% of cases, RSVC and in 35% of cases, the innominate vein are present [5]. In our case, RSVC was present but the left innominate vein was not.

Diagnosis of PLSVC is usually an incidental finding during cardiac surgery for retrograde cardioplegia, left subclavian vein cannulation for theuropathic or monitoring purposes [9, 10], device implantation [17] or cardiovascular imaging [4-6]. It is usually asymptomatic and clinically silent without additional congenital heart defects. But it may be symptomatic in patients with a PLSVC draining to left atrium that causes a hemodinamically significant right to left shunt with a variable degree of systemic cyanosis and clubbing in the absence of a RSVC [22]. In addition, some researchers suggested that patients with a PLSVC may become symptomatic due to arrhythmias through stretching of the atrioventricular node or His bundle by the dilated coronary sinus [23].

The standard technique for the diagnosis is echocardiography, either transthoracic or transoesophageal. The most common echocardiograpic finding of a PLSVC is a dilated coronary sinus and should prompt the echocardiographer to look for a PLSVC as well as for other etiologies such as elevated right atrial pressure (most common), coronary arterio-venous fistula, partial anomalous pulmonary venous return or unroofed coronary sinus. The diagnosis is then confirmed by contrast echocardiography. After agitated saline injection into a left-sided brachial vein, bubble contrast appears in the coronary sinus before appearing in the right atrium and ventricle. When agitated saline is injected into a right-sided brachial vein, the echo contrast enhances the right atrium before the coronary sinus, thus confirming a normal right superior vena cava [5]. It is also possible to diagnose PLSVC by radionuclide angiocardiography [24], computed tomography [6], and magnetic resonance imaging [25], although all of which are not cost-effective techniques and should be kept for exceptional cases.

PLSVC has important clinical implications in certain situations. During cardiac surgery, administration of retrograde cardioplegia is hampered by the presence of a persistent left superior vena cava, which results in excessive runoff of solution into the persistent left superior vena cava and the right atrium [26]. When the left subclavian vein is used for access, serious complications such as arrhythmia, cardiogenic shock, cardiac tamponade and coronary sinus thrombosis may occur as a result of manipulating the catheter in the CS [27]. Permanent pacemaker or implantable cardioverter defibrillator placement can be challenging.

In conclusion, because there is a significant increase in invasive cardiovascular and electrophsysiological procedures, special attention should be paid to this anatomical configuration. Lack of knowledge can pose serious complications. Transthoracic contrast echocardiography is the method of choice in the diagnosis of persistent left superior vena cava.
